# Modulatory Effects of Prediction Accuracy on Electroencephalographic Brain Activity During Prediction

**DOI:** 10.3389/fnhum.2021.630288

**Published:** 2021-02-25

**Authors:** Kentaro Ono, Junya Hashimoto, Ryosuke Hiramoto, Takafumi Sasaoka, Shigeto Yamawaki

**Affiliations:** ^1^Center for Brain, Mind, and KANSEI Sciences Research, Hiroshima University, Hiroshima, Japan; ^2^Graduate School of Education, Hiroshima University, Hiroshima, Japan; ^3^Center of Kansei Innovation, Hiroshima University, Hiroshima, Japan

**Keywords:** prediction, electroencephalography (EEG), stimulus preceding negativity (SPN), late positive components (LPCs), prediction error

## Abstract

Prediction is essential for the efficiency of many cognitive processes; however, this process is not always perfect. Predictive coding theory suggests that the brain generates and updates a prediction to respond to an upcoming event. Although an electrophysiological index of prediction, the stimulus preceding negativity (SPN), has been reported, it remains unknown whether the SPN reflects the prediction accuracy, or whether it is associated with the prediction error, which corresponds to a mismatch between a prediction and an actual input. Thus, the present study aimed to investigate this question using electroencephalography (EEG). Participants were asked to predict the original pictures from pictures that had undergone different levels of pixelation. The SPN amplitude was affected by the level of pixelation and correlated with the subjective evaluation of the prediction accuracy. Furthermore, late positive components (LPC) were negatively correlated with SPN. These results suggest that the amplitude of SPN reflects the prediction accuracy; more accurate prediction increases the SPN and reduces the prediction error, resulting in reduced LPC amplitudes.

## Introduction

Prediction of future events plays an important role in everyday life; we often predict situations to organize our behavior in preparation for the effects of those events. For example, when we drive a car and reach an intersection, we predict whether someone may run out into the street. Owing to the prediction process, the car can be stopped to prevent an accident. Psychological studies have shown that such predictions are beneficial for perceiving future stimuli that meet these predictions (Oliva and Torralba, 2007; Pinto et al., 2015; Stein and Peelen, 2015).

The neural dynamics underlying prediction have frequently been studied using electroencephalography (EEG) with an S1–S2 paradigm, which comprises the presentation of a cue (S1) followed by a target stimulus (S2). EEG studies have shown that the brain potential at the fronto-central area shifts toward the negative direction between S1 and S2, even though it initially shows positivity. This phenomenon is known as stimulus preceding negativity (SPN; Brunia et al., 2011a,b; Kotani et al., 2017). To date, the SPN has been hypothesized to be a physiological correlate of prediction and right hemisphere dominance since it originates from several brain regions, including the right insula, anterior cingulate cortex (ACC), et cetera (Böcker et al., 1994; Catena et al., 2012; Kotani et al., 2015). However, predictions are not always perfect, and we often organize our behavior with inaccurate predictions. If the SPN reflects prediction, it can be speculated that the amplitude of the SPN would be correlated with the accuracy of the prediction. However, only a few studies have focused on the accuracy of prediction using sentence comprehension (León-Cabrera et al., 2017, 2019), whereas many studies have investigated whether the uncertainty of S2 occurrence, as represented by S1, affects SPN (Kotani and Aihara, 1999; Hillman et al., 2000; Brown et al., 2008; Foti and Hajcak, 2012; Fuentemilla et al., 2013; Morís et al., 2013; Seidel et al., 2015; Novak et al., 2016). In these studies, participants received S1 as a cue that informed them of the occurrence of S2. However, the effects of uncertainty were not consistent among these studies. While some studies found a negative correlation between S2 uncertainty and SPN amplitude (Catena et al., 2012; Fuentemilla et al., 2013; Morís et al., 2013), others have found either no or positive correlation between them (Kotani and Aihara, 1999; Hillman et al., 2000; Brown et al., 2008; Seidel et al., 2015; Tanovic et al., 2018; Johnen and Harrison, 2020). The primary aim of the present study was to clarify how prediction accuracy affects SPN; this study used an S1–S2 paradigm with different levels of pixelated pictures as S1 and the original pictures as S2. We expected that different levels of pixelation would cause different levels of prediction accuracy for S2, leading to a difference in SPN amplitude.

Prediction is also a key component of predictive coding theory, which views the brain as a “predictive machine” (Friston et al., 2006; Clark, 2013). According to this theory, the brain continuously generates and updates predictions regarding an upcoming sensory input based on the principles of Bayesian inference. When the input does not match the prediction, a prediction error occurs. The error signal is projected upwards in the hierarchical neural network to update the prediction. This mechanism makes it possible to adapt our behavior to react to changes in the environment. A recent view of predictive coding suggested that attention to the stimuli increases the gain of neurons sending the prediction error to a higher level of hierarchical inference structure (Friston, 2009; Feldman and Friston, 2010). If this assumption is true, a highly pixelated S1 (blurred picture) can cause a larger prediction error than a slightly pixelated S1 (clear picture); this is because a highly pixelated S1 needs more attentional resources to predict the original picture. Since it is known that unpredicted stimuli cause larger late positive components (LPC), such as P300 or late positive potential (LPP; Delplanque et al., 2005; Gole et al., 2012; Chennu et al., 2013; Lin et al., 2015; Yang et al., 2019), the secondary aim of the present study was to clarify how prediction accuracy affects post-prediction neural responses. We expect that different levels of pixelation will cause different levels of prediction error, resulting in differences in the LPC.

We hypothesized that: (1) the accuracy of prediction is associated with SPN; and that (2) the accuracy of prediction is negatively associated with the LPC. These hypotheses were evaluated using an S1–S2 paradigm, comprising pixelated pictures (S1) and original pictures (S2). Different degrees of pixelation were used to change the accuracy of prediction and we expected that the amplitude of SPN and LPC would be positively and negatively correlated with the accuracy of prediction, respectively.

## Materials and Methods

### Participants

Twenty-four healthy participants (10 men and 14 women) between 18 and 29 years of age [mean ± standard deviation (SD) = 22.7 ± 3.5] were enrolled in this study. All participants were right-handed, based on a Japanese version of the FLANDERS handedness questionnaire (Okubo et al., 2014). None of the participants reported motor, hearing, visual, or neurological deficits. All participants provided written informed consent to participate in the study. The experiment was performed as per the ethical standards of the Declaration of Helsinki and the guidelines were approved by the local ethics committee of Hiroshima University.

### Stimuli

The stimuli consisted of 240 colored pictures (120 positive and 120 negative) that were obtained from the Open Affective Standardized Image Set (OASIS; Kurdi et al., 2017). There were three steps for selecting pictures as stimuli from 900 pictures (500 × 400 pixels) in the OASIS database. First, we rejected 68 sexually explicit, violent, or traumatic pictures (e.g., dead body, nudity, or ugly wounds). Second, we selected 637 pictures with arousal in the neutral range (between 3 and 5 on a 7-point scale). Third, we rejected 160 pictures with a difference of more than two SD from the mean sharpness, spatial frequency (high and low), luminance, and colorfulness. After these selections, 293 positive and 184 negative pictures were obtained. Finally, to maximize the prediction error after the predictive phase, 120 positive and 120 negative pictures were selected as stimuli in order of valence.

**Figure 1:**
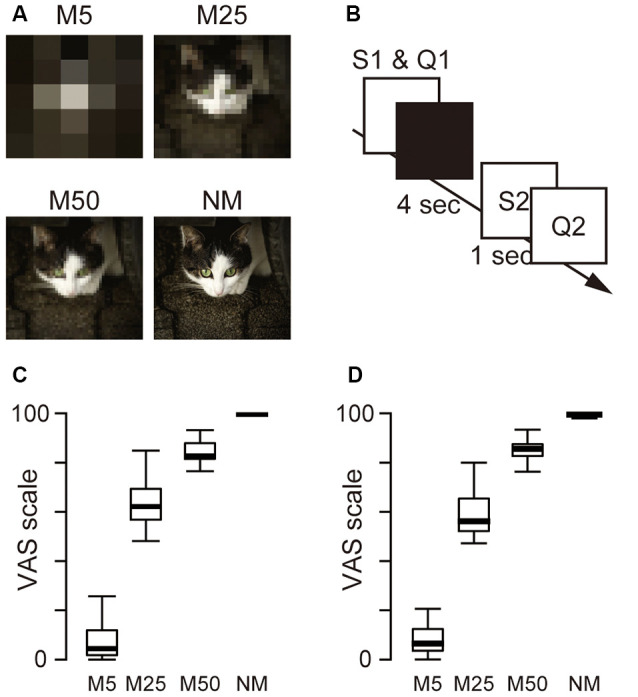
**(A)** Example of the picture stimuli used in the experiment. Three levels of pixelation [5 × 5 pixels (M5) 25 × 25 pixels (M25), 50 × 50 pixels (M50)] and no pixelation (NM; original picture) were used as S1. The original picture was also used as S2. **(B)** Schematic presentation of a single trial. S1 stimulus and Q1 (how confident the participant felt about the prediction of S2) were presented simultaneously until the participants responded. The monitor then turned black for 4 s for the predictive phase. Subsequently, Q2 (how accurate the predicted image and S2 matched) was presented after a 1-s presentation of S2. **(C)** The box plot of the mean score of Q1. **(D)** The box plot of the mean score of Q2.

To pixelate the pictures, the original pictures were divided into tiles [5 × 5 mosaic (M5), 25 × 25 mosaic (M25), and 50 × 50 mosaic (M50)] and the RGB values in each tile were averaged. M5 resulted in the lowest picture quality, and M50 produced the highest quality. In total, 960 pictures, including 240 original (no pixelation) and pictures with three different levels of pixelation (M5, M25, and M50), were used as stimuli in the experiment. This procedure was performed using MATLAB (version R2017b, MathWorks, USA). Examples of the pixelated and original pictures are shown in Figure 1A. These stimuli were presented using Presentation (ver. 20.0, Neurobehavioral Systems) on a Dell Workstation (Dell Precision Tower 5810, Dell Inc., Round Rock, TX, USA).

### Experimental Design

Participants were seated in a chair in a dimly lit soundproof room. The visual stimuli were presented on a dark background at the center of a 24-inch display (U2412M, 1,920 × 1,200 pixels, 60 Hz, Dell, USA) placed at a distance of ~70 cm from the participant (subtending a visual angle of ~2°). During the EEG measurement, the participants performed 240 trials of two subjective evaluation tasks (Q1 and Q2) in six blocks in an S1–S2 paradigm (40 trials in each block). Q1 asked the participant’s confidence in the prediction after watching a pixelated picture. This question was used to evaluate the accuracy of the participants’ predictions. Q2 asked the difference between the prediction and the original picture to evaluate the size of the difference between the predicted and actual inputs. In each trial (Figure 1B), the participants viewed one of the four kinds of picture stimuli [M5, M25, M50, and the original picture as control (No Mosaic; NM)] as S1, and were asked to predict what the original picture was. Participants then evaluated their confidence in their prediction (Q1) and were asked to keep predicting the original picture for 4 s (predictive phase). After the predictive phase, the original picture (S2) appeared, and 1 s later, they began to evaluate the accuracy of prediction (Q2). Participants answered two questions (Q1 and Q2) using a visual analog scale (VAS) ranging from 0 to 100. To answer the questions, they moved the PC mouse left or right using their right hand and clicked to indicate the value on the VAS scale.

### EEG Measurement

The EEG was sampled at 1,000 Hz using a BrainAmp DC amplifier (Brain Products GmbH, Munich, Germany) with 64 channel actiCAP electrodes. The electrodes were placed according to an extended international 10-10 system. The ground electrode was placed on the forehead (position: Fpz), and the EEG signal was recorded using a nose reference. To evaluate artifacts caused by eye movements, electrooculograms (EOGs) were recorded with electrodes placed above and below the left eye (vertical EOG) and beside both eyes (horizontal EOG). The electrode-skin impedance was maintained below 20 kΩ. The participants had their heads immobilized on a chin rest to avoid movement artifacts.

### Data Analysis

EEG data were analyzed using EEGLAB (Delorme and Makeig, 2004). After down-sampling to 500 Hz and filtering with a bandwidth of 0.1–40 Hz, an epoch between 500 ms before and 6,000 ms following the response to Q1 was defined as the event-related potential (ERP) during the predictive phase. Besides, an epoch between 500 ms before and 1,000 ms after the onset of S2 was also defined as the ERP after prediction, a period believed to reflect the error detection process between a prediction and an incoming stimulus and an updating of the prediction. EOG artifacts were automatically removed from the data using blind source separation (BSS) included in the AAR plug-in (version 1.3) for EEGLAB (Gomez-Herrero et al., 2006). Subsequently, epochs with peak-to-peak amplitudes exceeding 200 μV were removed as artifacts. Following the exclusion criteria, approximately 6% of the trials were rejected. The epochs were then separately averaged for each electrode site. The SPN was defined as the amplitude of the brain potential observed in the frontal and central sites in a time window of 200 ms immediately before S2 presentation as used in previous studies (Brunia and Damen, 1988; Kotani and Aihara, 1999; Mattox et al., 2006; Catena et al., 2012; Kotani et al., 2015). The LPC was defined as the waveforms observed in Pz between 400 and 600 ms after S2 was presented.

### Statistical Analysis

The participants’ subjective evaluations (Q1 and Q2) were analyzed using a one-way analysis of variance (ANOVA) with a factor of pixelation (M5, M25, M50, and NM). The brain potentials observed in the frontal (F1 and F2), central (C1 and C2), and parietal (P1 and P2) areas were used to analyze the prediction phase. Regarding the SPN, a two-way ANOVA with the factors of the hemisphere (left and right) and pixelation was conducted for three separate areas. The brain potentials observed in the Pz after the predictive phase were analyzed using a one-way ANOVA with a factor of pixelation. A Greenhouse–Geisser correction was used to assess the sphericity. Simple main effect analysis was used to further evaluate interactions, and *post-hoc* multiple comparisons were conducted using paired *t-tests* with Shaffer’s modified sequentially rejective Bonferroni correction (Shaffer, 1986). Furthermore, Pearson’s product-moment correlation coefficient was estimated to evaluate the correlation between subjective evaluations and EEG data, and multiple comparisons were conducted using Bonferroni’s correction. Statistical analyses were performed using R studio (version 1.0.136) and R software (version 3.3.2; R Core Team, 2020).

## Results

Figures 1C,D also show box plots for Q1 and Q2. A one-way ANOVA for Q1 and Q2 with pixelation (M5, M25, M50, and NM) was conducted separately and showed the main effect (Q1: *F*_(3,23)_ = 248.9, *p* < 0.001; Q2: *F*_(3,23)_ = 1,779.0, *p* < 0.001), indicating that the level of confidence in the prediction (Q1) and the degree of accuracy of the prediction (Q2) were associated with the level of pixelation.

**Figure 2:**
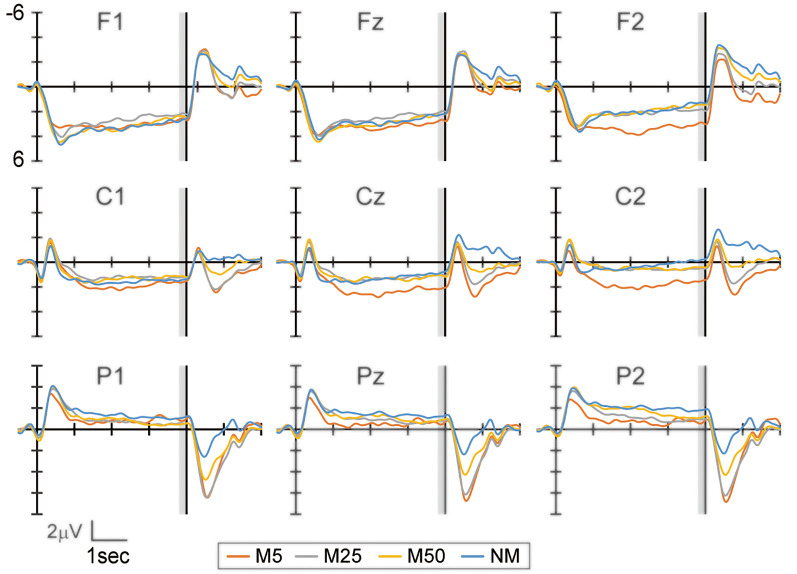
The event-related potential (ERP) waveform at the frontal (F1, Fz, and F2), central (C1, Cz, and C2), and parietal (P1, Pz, and P2) electrodes during the prediction phase (4 s). The start of the prediction phase was 0 ms. The time window with gray color (3,800–4,000 ms) was used to analyze the stimulus preceding negativity (SPN) amplitude. Note that the waveforms in this figure were filtered (1 Hz low-pass) for visualization purposes only.

Figure 2 shows the EEG waveform during the prediction phase in the frontal (F1, Fz, and F2), central (C1, Cz, and C2), and parietal areas (P1, Pz, and P2). The bilateral frontal electrodes showed a positive peak at approximately 500–1,000 ms after the response to Q1. Subsequently, this peak gradually decreased until the S2 presentation. The bilateral central electrodes showed a negative peak around 500–1,000 ms, which moved to positive before 1,000 ms. This peak then remained positive during the prediction phase. In contrast, the parietal electrodes showed a negative peak and became smaller until the S2 presentation. While the frontal electrode showed positive potential during the prediction phase, the central electrodes reversed the polarity from negative to positive. This might reflect the motor preparation in response to Q2. To evaluate the effect of pixelation level on SPN, the mean amplitude of the brain potential in a time window of 200 ms immediately before S2 presentation was analyzed using two-way ANOVA, with factors of the hemisphere (left and right) and pixelation level (M5, M25, M50, and NM) for the frontal, central, and parietal areas separately (frontal: F1 and F2, central: C1 and C2, and parietal: P1 and P2).

**Figure 3:**
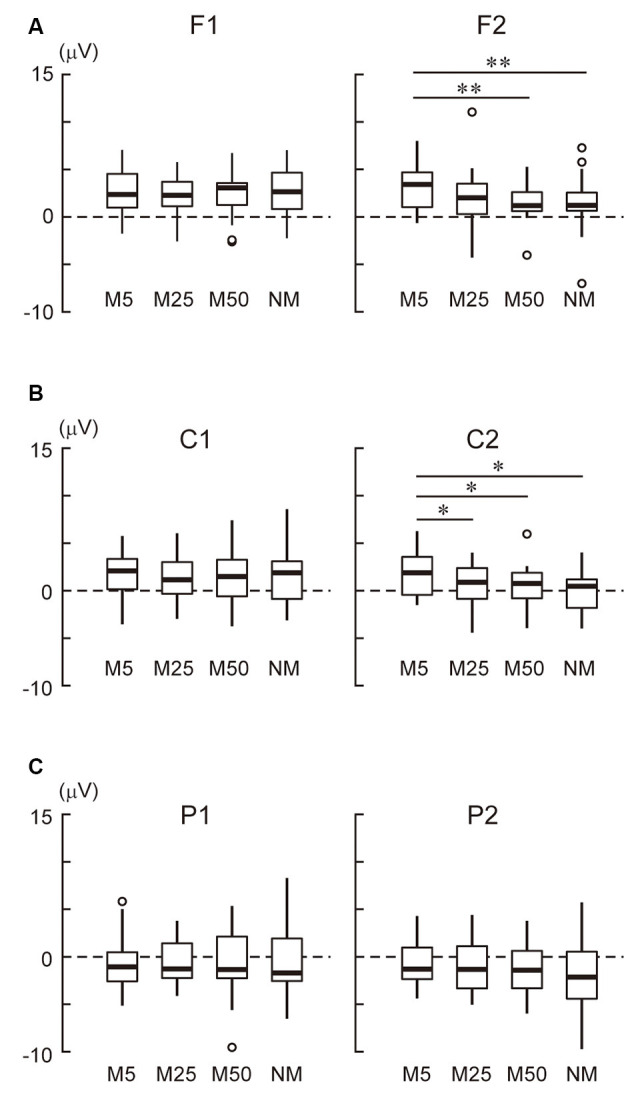
**(A)** The mean amplitude of the stimulus preceding negativity (SPN) at the frontal area (F1 and F2). **(B)** The mean amplitude of the SPN at the central area (C1 and C2). **(C)** The mean amplitude of the SPN at the parietal area (P1 and P2). Note that the positive values were in an upwards direction. A white circle indicates an outlier. * and ** indicate *p* < 0.05 and *p* < 0.01, respectively.

In the frontal area (Figure 3A), the ANOVA only showed a two-way interaction (*F*_(3,69)_ = 6.50, *p* < 0.001). A simple effect analysis showed that the effect of pixelation level was significant in the right hemisphere (F2 electrode: *F*_(3,69)_ = 5.34, *p* = 0.002). Multiple comparisons revealed that M5 was more positive than M50 (*t*_23_ = 4.12, adjusted *p* = 0.003) and NM (*t*_23_ = 3.62, adjusted *p* = 0.004). In the central area (Figure 3B), the ANOVA showed the main effects of hemisphere (*F*_(1, 23)_ = 6.44, *p* = 0.018) and pixelation level (*F*_(3,69)_ = 3.54, *p* = 0.019), as well as an interaction (*F*_(3,69)_ = 4.74, *p* = 0.005). A simple effect analysis showed that the effect of pixelation was significant in the right hemisphere (C2 electrode: *F*_(3,69)_ = 5.96, *p* = 0.001). Multiple comparisons revealed that M5 was more positive than the others (M25: *t*_23_ = 2.72, adjusted *p* = 0.037; M50: *t*_23_ = 2.84, adjusted *p* = 0.028; NM: *t*_23_ = 3.29, adjusted *p* = 0.019). In the parietal area (Figure 3C), ANOVA only showed an interaction (*F*_(3,69)_ = 2.87, *p* = 0.042). A simple effect analysis showed that the effect of pixelation level was significant in the right hemisphere (P2 electrode: *F*_(3,69)_ = 2.81, *p* = 0.046). However, multiple comparisons did not show a significant difference between pixelation levels. These results indicate that the roughest mosaic (M5) elicited more positive potentials in the right fronto-central area.

**Figure 4:**
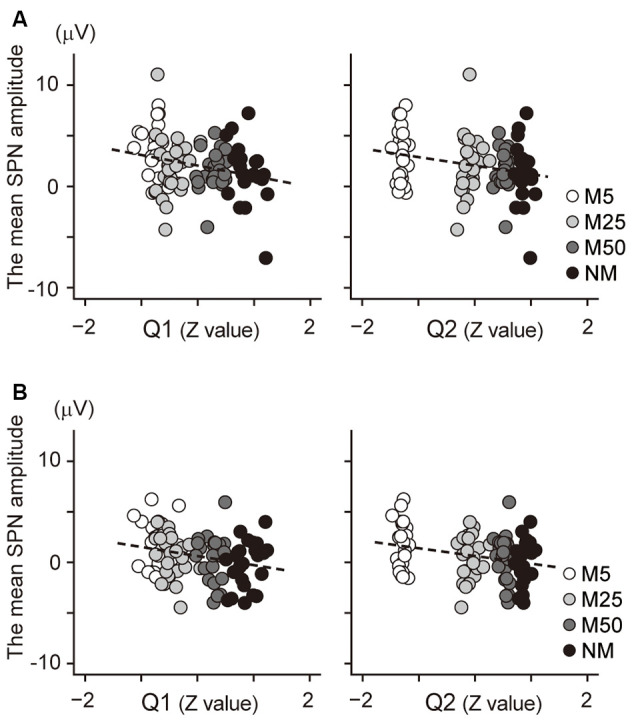
**(A)** Scatter plot to show the correlation between the SPN amplitude at F2 electrode and the normalized value of the Q1 score (*left*) and the Q2 score (*right*). **(B)** A scatter plot to show the correlation between the SPN amplitude at the C2 electrode and the normalized value of the Q1 score (*left*) and the Q2 score (*right*). The regression line is represented by a dashed line.

Correlation analysis was conducted to investigate the relationship between the SPN and the subjective confidence of the prediction. The mean amplitude of the brain potential at the F2 electrode (Figure 4A) showed a negative correlation with the Q1 score (confidence in the prediction: *r* = −0.25, *t*_94_ = −2.55, *p* = 0.024), indicating that a higher Q1 score was associated with more negative potential at F2; this negative potential indicates a larger SPN. Thus, this result suggests that a higher Q1 score is associated with a larger SPN. Besides, the brain potential at F2 was also negatively correlated with the Q2 score (the accuracy of prediction: *r* = −0.25, *t*_94_ = −2.56, *p* = 0.024), indicating that higher Q2 scores were associated with larger SPN. The correlation between the brain potential at the C2 electrode and Q1/Q2 score also showed a positive correlation (Figure 4B; Q1: *r* = −0.25, *t*_94_ = −2.48, *p* = 0.030; Q2: *r* = −0.28, *t*_94_ = −2.79, *p* = 0.012). Taken together, these results indicate that the SPN amplitudes at F2 and C2 were associated with confidence in the prediction (Q1) and accuracy of prediction (Q2).

**Figure 5:**
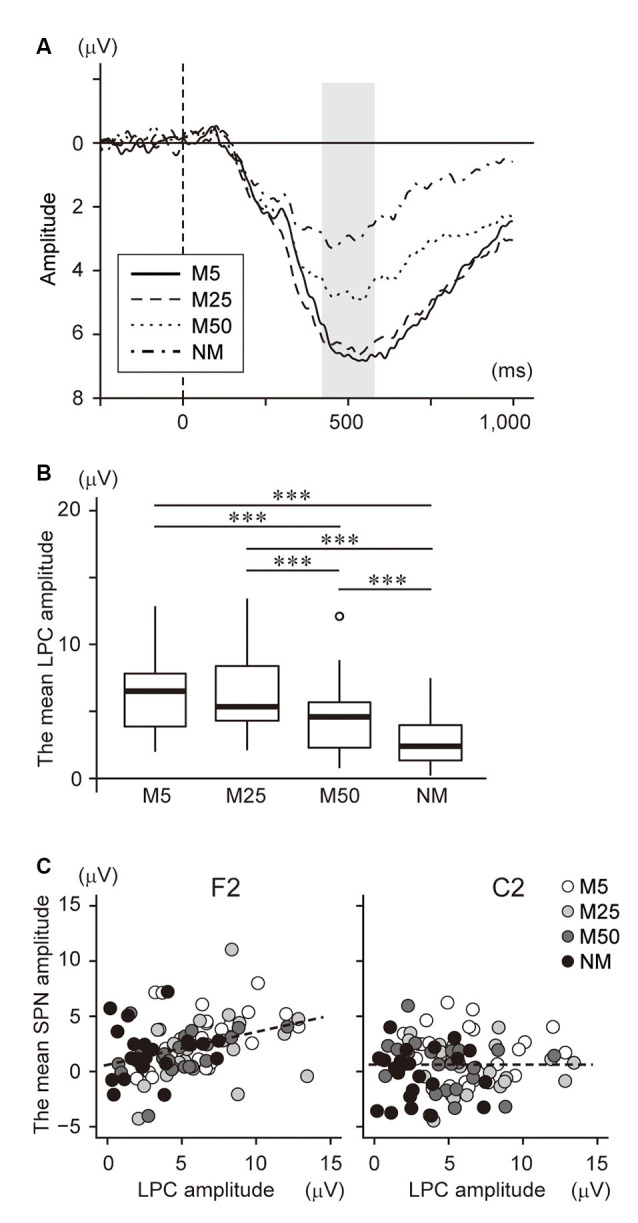
**(A)** The event-related potential (ERP) waveform at Pz after S2 presentation. The onset of S2 presentation was 0 ms. The time window with gray color (400–600 ms) was used to analyze the late positive components (LPC) amplitude. **(B)** The mean amplitude of the LPC at Pz. **(C)** Scatter plot to show the correlation between the LPC amplitude and the SPN at F2 (*left*) and C2 (*right*). ***Indicates *p* < 0.001. The regression line is represented by a dotted line.

We also investigated the relationship between SPN and brain responses during the post-prediction phase. First, the waveform at Pz was plotted in Figure 5A because it is known that LPC, including P300 and LPP, are often observed in the parietal area and reflect the prediction error. This shows a large positive potential with a peak of around 400–600 ms. The mean amplitude during this time window was analyzed using one-way ANOVA with a factor of pixelation level (Figure 5B). This analysis showed a main effect (*F*_(3,69)_ = 36.78, *p* < 0.001). Multiple comparisons revealed that the amplitude for NM was smaller than for all others (NM < M5: *t*_23_ = 6.78, *p* < 0.001; NM < M25: *t*_23_ = 8.08, *p* < 0.001; NM < M50: *t*_23_ = 4.31, *p* < 0.001). The LPC amplitude of M50 was also smaller than that of M5 (*t*_23_ = 5.52, *p* < 0.001) and M25 (*t*_23_ = 6.25, *p* < 0.001). Furthermore, correlation analysis between the LPC amplitude and the accuracy of prediction (Q2) showed a significant negative correlation (*r* = −0.39, t_94_ = −4.10, *p* < 0.001). These results indicated that the LPC was negatively associated with the pixelation level and accuracy of prediction.

To investigate the interaction between SPN and LPC, we conducted correlation analysis between the brain potentials at F2 and C2 and the LPC at Pz (Figure 5C), which showed a significant positive correlation with the brain potential at F2 (*r* = 0.38, *t*_94_ = 4.00, *p* < 0.001), while no significant correlation was found at C2 (*r* = 0.001, *t*_94_ = 0.01, *p* = 0.99). Note that a negative potential at F2/C2 indicates a larger SPN. Thus, this result suggests that a larger SPN is associated with smaller LPC.

## Discussion

In the present study, we used electrophysiological recordings to elucidate the influence of prediction accuracy on brain activity during the prediction of an upcoming stimulus. We hypothesized that the accuracy of the prediction was associated with the amplitude of the SPN, and negatively associated with the amplitude of the LPC. Consistent with this hypothesis, we obtained the following three main findings. First, the SPN amplitude was smallest when the quality of S1 was the lowest. Second, the SPN amplitude at the right frontal and central sites was correlated with the participant’s subjective evaluation of confidence in the prediction (Q1) and accuracy of the prediction (Q2). Finally, the SPN amplitude at F2 increased as the LPC amplitude decreased.

The first finding supports those of previous studies suggesting that the SPN is an index of predictive processes in the brain (Brunia, 1988; Kotani et al., 2017). Although our findings did not show a gradual increase in the SPN amplitude according to the level of pixelation, this is in agreement with the results of previous studies, which showed that the SPN did not reflect different degrees of S2 uncertainty (Tanovic et al., 2018; Johnen and Harrison, 2020). These studies suggest that the SPN may index anticipatory processes based on a coarser appraisal of whether an upcoming event is either certain or uncertain to occur. The first finding may also reflect similar processes. However, an interesting difference between the previous studies and this study is the second finding that we found a significant correlation between the SPN amplitude and Q1/Q2 scores. Together with the first finding, these results suggest that the SPN amplitude reflects the subjective evaluation of prediction (confidence and accuracy), but not the physical properties of the stimulus (probability of occurrence and level of pixelation).

Also, the second finding suggests right hemispheric dominance regarding the occurrence of SPN. Participants reported very low scores at Q1/Q2 in the condition with the greatest pixelation (M5), indicating that it was almost impossible to predict S2. Conversely, they also reported high scores at Q1/Q2 in the least pixelation (M50) and control (NM) conditions, indicating that it was easy to predict S2. In these cases, the participants’ intrinsic motivation should be different, corresponding to the confidence and accuracy of the prediction. In general, if the difficulty of a task is high and perceived as impossible or beyond one’s control, intrinsic motivation should be low. Motivational intensity depends on the perceived difficulty of a task (Brehm and Self, 1989), and neural circuits supporting motivational processing, including the brain areas, such as the insula and thalamus, are activated during prediction (Knutson and Greer, 2008; Ivanov et al., 2012). Also, fMRI studies have found that the right insula is correlated with participants’ intrinsic satisfaction in performing experimental tasks (Lee et al., 2012; Lee and Reeve, 2013). This evidence suggests that the activity in the right insula was not only associated with the prediction accuracy, but also with the participant’s motivational intensity. Thus, right hemisphere dominance can be interpreted as a consequence of the increase in intrinsic motivation to accurately predict S2. This caused additional activation of the right insula, appearing as a Q1/Q2 score-dependent change in SPN in the right hemisphere.

The influence of motivation might also help to interpret inconsistent results reported by previous studies on SPN. While some studies have found a negative correlation between the probability of S2 occurrence and SPN amplitude (Catena et al., 2012; Fuentemilla et al., 2013; Morís et al., 2013), other studies have found no or positive correlation between them (Kotani and Aihara, 1999; Hillman et al., 2000; Brown et al., 2008; Seidel et al., 2015). If the probability of S2 occurrence is low, some participants might find it difficult to maintain their motivation to perform the task, while other participants could maintain motivation. Thus, the inconsistency might be based on a difference in intrinsic motivation between certain and uncertain conditions.

The prediction accuracy also influenced post-prediction brain processing. The recent view of predictive coding suggests that attention increases neuronal gain to cause prediction errors. Based on this idea, the third finding can be interpreted as follows: in the present study, more attention was needed to create a prediction by rough S1 (M5 or M25) because of the lack of information. Although many attentional resources were applied, it was almost impossible to create an accurate prediction using a rough S1. In contrast, it was easy to create an accurate prediction using a clear S1, such as M50 or NM, with fewer attentional resources. In this case, the prediction accuracy for a rough S1 was small, but the attentional gain was large; conversely, the prediction accuracy for a clear S1 was large, but the attentional gain was small. Thus, it is expected that prediction accuracy is negatively associated with attentional gain. From the predictive coding, a large attentional gain increases the prediction error, and the third finding that the SPN amplitude was negatively correlated with the LPC, validated this argument.

This interpretation is supported by the negative correlation between the LPC and Q2 (accuracy of prediction). Because the Q2 score can be interpreted as negatively associated with the prediction error, the negative correlation between the LPC and Q2 indicates that the LPC amplitude is positively correlated with the prediction error. Although it is difficult to clearly separate LPC as the P300 and LPP components in the present study, this finding is consistent with those of previous studies that showed that a brain potential of approximately 300–600 ms in an uncertain situation indicates the violation of prediction (Mangun and Hillyard, 1991; Delplanque et al., 2005; Philiastides et al., 2010; Johnen and Harrison, 2019). However, several different explanations have been proposed regarding cognitive processes associated with the brain potential in this time window, especially the LPP, such as memory formation, emotional processing, attentional modulation, or motivational salience (Olofsson et al., 2008; Foti et al., 2009; Hajcak et al., 2010). Also, the LPC may include the P300 components. P300 can be separated as P3a, usually elicited by infrequent distractor stimuli in the fronto-central area, and P3b, elicited by a target stimulus to respond in the parietal area (Polich, 2007). The LPCs observed in this study might include P3b and LPP components. However, this is speculation because we could not separate the LPCs in the design of our experiment. Therefore, further verification studies are warranted.

While the SPN is thought to originate from the brain areas including the right insula, the prediction error is coded in diverse regions throughout the cerebral cortex, striatum, and the medial frontal structures, such as the ACC (Garrison et al., 2013). Predictive coding theory hypothesizes that prediction and sensory inputs are coded in separate neural populations within the cortical areas. Based on this idea, the present findings suggest that a prediction generated in prediction-related brain areas, including the right insula, will be sent to the sensory areas to compare the prediction with the actual input. This comparison caused a certain amount of prediction error, and the error signal was sent to the prediction error network to elicit the LPC. Unfortunately, it is difficult to conduct a detailed source analysis of SPN and LPC using EEG. Thus, we can only speculate that such a diverse brain network might process the prediction and prediction errors. Thus, further research is required to clarify this issue.

In summary, the present study investigated the effect of prediction accuracy on brain processes during and after prediction. During the prediction of original pictures from pixelated pictures, the SPN was found in the right fronto-central area to correlate with the subjective evaluation of the prediction accuracy. Also, the SPN in the right frontal area correlated with ERP following the presentation of the original pictures. After the prediction phase, the LPC was found in the parietal area and negatively correlated with the subjective evaluation of the prediction accuracy and SPN amplitude. These results suggest that certain predictions increase the activity in the prediction-related brain areas, leading to a large SPN, and the difference between the prediction and the actual input activates the prediction error network, leading to large LPC. According to the predictive coding theory, these networks include separate neural populations. This should be clarified in future studies.

## Data Availability Statement

The raw data supporting the conclusions of this article will be made available by the authors, without undue reservation.

## Ethics Statement

The studies involving human participants were reviewed and approved by the local ethics committee of Hiroshima University. The patients/participants provided their written informed consent to participate in this study.

## Author Contributions

KO and TS conceived and KO, TS, and SY designed the experiment. KO, JH, and RH contributed the data acquisition and analysis. KO wrote the manuscript and all authors reviewed the manuscript. All authors contributed to the article and approved the submitted version.

## Conflict of Interest

The authors declare that the research was conducted in the absence of any commercial or financial relationships that could be construed as a potential conflict of interest.
